# A Rare Case of Gorlin-Goltz Syndrome in Children

**DOI:** 10.1155/2019/1608783

**Published:** 2019-12-23

**Authors:** Fernanda Brasil Daura Jorge Boos Lima, Ana Paula Cota Viana, Luciano Henrique Ferreira Lima, Bruna Campos Ribeiro, Carlos Eduardo Assis Dutra, Glaykon Alex Vitti Stabile, Sergio Monteiro Lima Junior

**Affiliations:** ^1^Department of Clinics, Pathology and Surgery, Federal University of Minas Gerais, Brazil; ^2^Graduation in Dentist, Department of Dentistry, PUC Minas, Brazil; ^3^Graduation in Dentistry, Federal University of Minas Gerais, Brazil; ^4^Department of Dental Medicine, State University of Londrina, Brazil

## Abstract

The Gorlin-Goltz syndrome, nevoid basal cell carcinoma syndrome, or basal cell nevus syndrome is an autosomal dominant condition disorder with high variability expression. It presents a series of relevant clinical manifestations that suggest its diagnosis in cutaneous, bone, dental, soft tissue, nervous, and ocular system disorders. This condition requires a great interaction of several specialists to improve the patient's life. In this case, we presented a 9-year-old male patient referred to the Department of Oral and Maxillofacial Surgery reporting failure in the normal chronology of dental eruption. After evaluation, it was observed that the patient had 13 typical characteristics of the syndrome, including keratocysts, bifid ribs, palmoplantar pits, and 10 other minor characteristics. In conclusion, the expression of so many features of Gorlin-Goltz syndrome is rare in infants, and early diagnosis is important to decrease morbidity and mortality associated with basal cell carcinomas.

## 1. Introduction

The Gorlin-Goltz syndrome or nevoid basal cell carcinoma syndrome was first described in 1894 by Jarish and White, being an autosomal dominant condition with high expression variability. In the year of 1960, Gorlin and Goltz [1–3] described a series of basal cell carcinomas, odontogenic keratocysts, and bifid ribs including new clinical characteristics to the syndrome.

The diagnostic criteria for the Gorlin-Goltz syndrome were divided into major and minor, the first being composed of the presence of more than two basal cell carcinomas or a history of one basal cell carcinoma below the age of 20 years; odontogenic keratocysts of the jaw (histologically confirmed); three or more palmoplantar pits; bifid, fused, or markedly splayed ribs; and presence of a diagnosis of the Gorlin-Goltz syndrome in a first-degree relative. The minor criteria are falx cerebri calcification, macrocephaly, congenital anomalies, cleft lip palate, frontal bossing, coarse face, hypertelorism, skeletal anomalies (rib anomalies, kyphosis/scoliosis, shortened fourth metacarpal, polydactyly/syndactyly, radiolucencies/pseudocysts in the hands or feet, sloping shoulders, and immobile thumbs), neurologic or central nervous system anomalies (medulloblastoma, meningioma, agenesis of the corpus callosum, congenital hydrocephalus, and intellectual disability), impacted or ectopic teeth, bilateral coronoid hyperplasia and oligodontia sprengel deformity, pectus deformity, bridging of the sella turcica, hemivertebra and combined vertebral corpi, ovarian fibroma, and medulloblastoma [1, 4, 5]. The correct diagnosis of the Gorlin-Goltz syndrome is obtained by the presence of two major criteria or two minor and one major criteria [2].

Basal cell carcinomas are usually diagnosed after puberty. Due to the susceptibility to the development of neoplasm and the syndrome becoming very aggressive as age progresses, the diagnosis should be precocious [1]. This is the first report of a child with so many characteristics of this syndrome that has not presented skin carcinomas yet. The early diagnosis is important to decrease morbidity and mortality associated with basal cell carcinomas.

## 2. Case Report

A 9-year-old male patient was referred to the Department of Oral and Maxillofacial Surgery due to failure of dental eruption in many teeth. The patient presented multiple cysts in the mandible and maxilla with a diagnostic hypothesis of odontogenic keratocysts.

**Figure 1:**
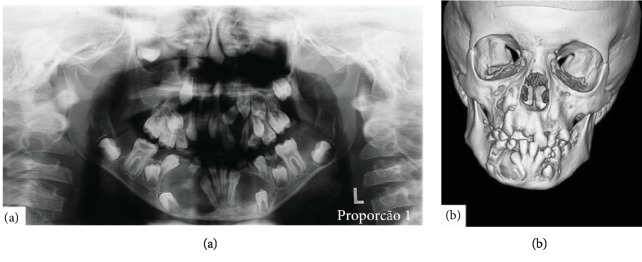
(a) Panoramic radiograph showing the multiple odontogenic keratocysts in the maxilla and mandible. (b) Computed tomography of the jaw.

The intraoral examination revealed several dental absences at the anterior and posterior regions of the maxilla and mandible, with teeth in the ectopic position, beside the bulging of the vestibular cortical bone in the region of the right inferior canine and premolars. On the panoramic radiograph, multiple radiolucent areas, causing dental dislocation, were observed (Figure 1(a)). With the objective of obtaining the most precise limits of the multiple lesions, computed tomography was done (Figure 1(b)).

**Figure 2:**
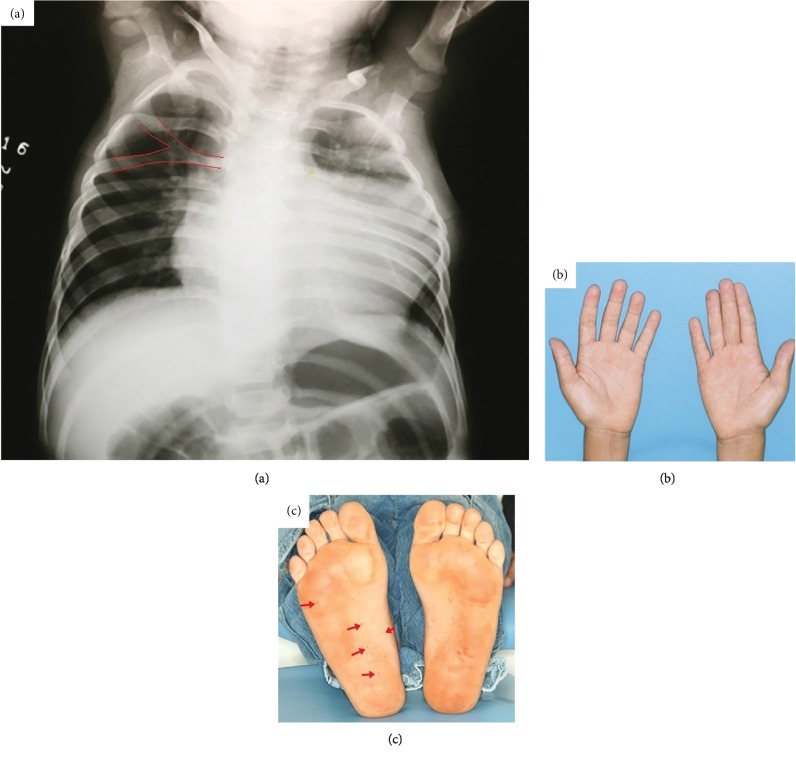
(a) Bifid rib; (b, c) palmoplantar pits.

**Figure 3:**
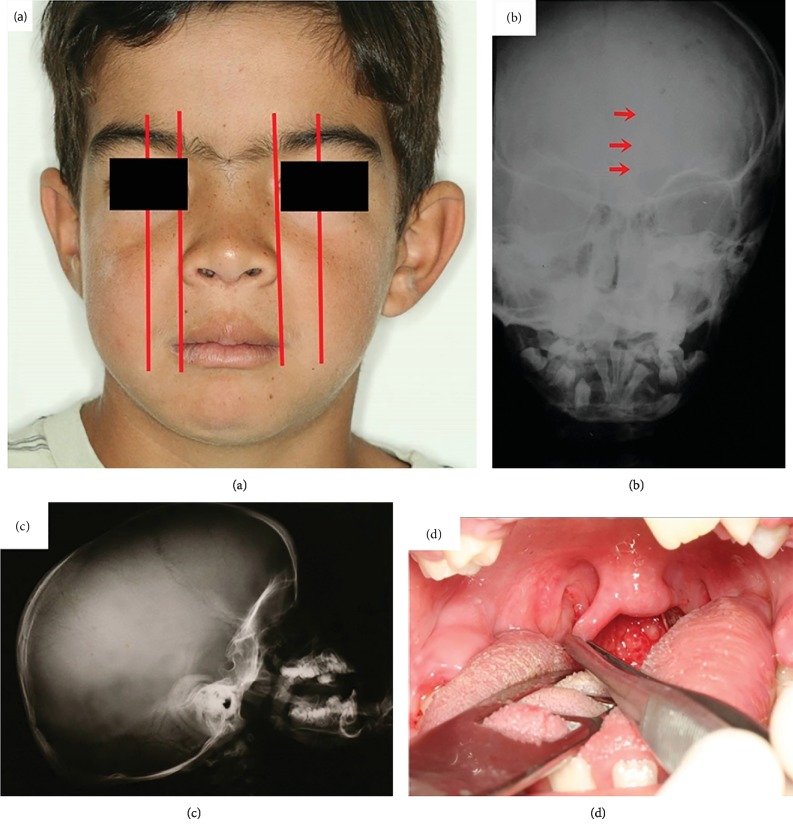
(a) Hypertelorism; (b) falx cerebri calcification; (c) macrocephaly; (d) bifid uvula.

**Figure 4:**
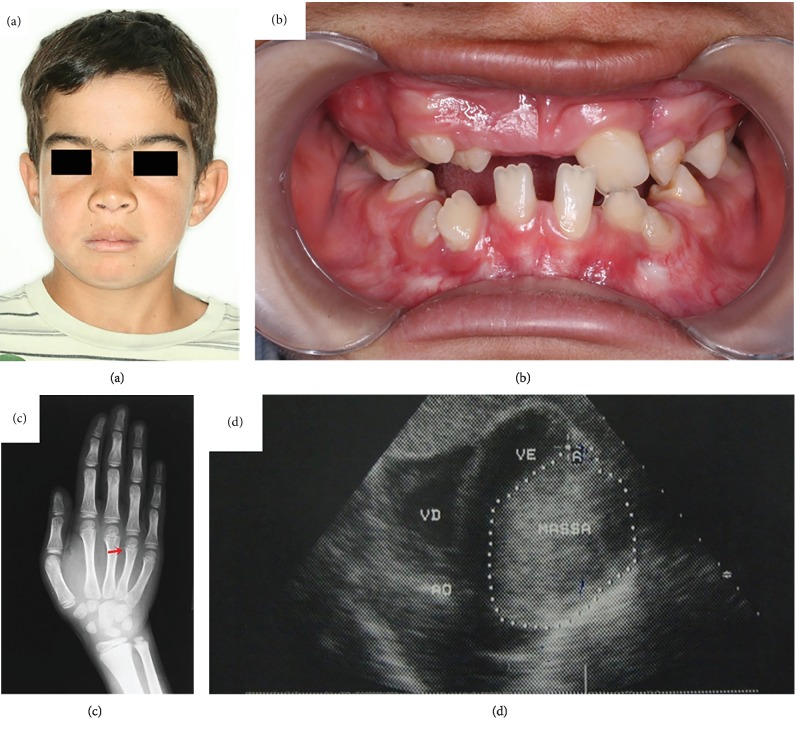
(a) Frontal bossing; (b) malocclusion; (c) shortened fourth metacarpal; (d) cardiac fibroma.

Odontogenic keratocysts were histologically confirmed and treated by marsupialization, enucleation, and follow-up. Due to the possibility of multiple keratocysts being associated with Gorlin-Goltz syndrome, other manifestations of the Gorlin-Goltz syndrome were searched. After this clinical evaluation, three major criteria were found: odontogenic keratocysts (Figure 1), bifid ribs (Figure 2(a)), and palmoplantar pits (Figures 2(b) and 2(c)), along with 10 minor criteria: hypertelorism (Figure 3(a)), falx cerebri calcification (Figure 3(b)), macrocephaly (Figure 3(c)), bifid uvula (Figure 3(d)), frontal bossing (Figure 4(a)), malocclusion (Figure 4(b)), shortened fourth metacarpal (Figure 4(c)), cardiac fibroma (Figure 4(d)), ectopic dental position, and precocious development of genitals.

## 3. Discussion

In the present report, it is possible to verify some common findings within the literature; for example, the patient has leukoderma and he did not present a family-positive history. Among the unusual findings, it is possible to verify the manifestation of the characteristics of this syndrome in the first decade, considering that one of the primary manifestations detected is keratocysts, more frequent in the second to fifth decades of life. The recognition of this syndrome is a challenge, especially in children, because the majority of the major criteria are not present before the second or third decade of life [1, 6–8].

Manfredi et al. declares that the diagnosis of the Gorlin-Goltz syndrome initially consists in the triad of basal cell carcinomas, odontogenic keratocysts, and skeletal anomalies. In the present case, the diagnosis of odontogenic keratocysts was the first clinical manifestation of this condition. Thus, the importance of the recognition of this lesion by oral and maxillofacial health professionals is concluded [6].

The described patient had 13 characteristics that match with the diagnosis of this syndrome, being 3 major characteristics (odontogenic keratocysts in the maxilla and mandible, palmoplantar pits, and bifid ribs) and 10 minor characteristics (falx cerebri calcification, macrocephaly, frontal bossing, hypertelorism, cardiac fibroma, malocclusion, ectopic dental position, shortened fourth metacarpal, precocious development of the genitals, and bifid uvula). To the best of our knowledge, the literature did not mention a child with so many manifestations, this case being the first report.

In this case, the patient still had no basal cell carcinomas but attention is needed on the possible manifestation of these lesions, since this is also a characteristic of the syndrome and may be associated with a worse prognosis [9].

In this report, the treatment for the multiple odontogenic keratocysts consisted of marsupialization followed by enucleation. Long-term follow-up is mandatory, due to the high possibility of recurrence [9], because when the lesion is associated with the Gorlin-Goltz syndrome, rate of recurrence is 82%, due to the greater number of satellite cysts, solid islands of epithelial proliferation within the capsule, and mitotic figures in the epithelial lining of the main cavity [10, 11].

This case report stresses the importance of early diagnoses to prevent morbidity and mortality associated with this syndrome and reports a rare case of so many manifestations of the Gorlin-Goltz syndrome in an infant. In addition, the case report shows the importance of a multidisciplinary team including dentists, dermatologists, geneticists, and neurologists needed to improve the diagnosis and quality of life.
